# A201 EFFECT OF MIRIKIZUMAB ON BOWEL URGENCY CLINICALLY MEANINGFUL IMPROVEMENT AND REMISSION: RESULTS FROM THE PHASE 3 LUCENT INDUCTION AND MAINTENANCE STUDIES

**DOI:** 10.1093/jcag/gwac036.201

**Published:** 2023-03-07

**Authors:** S Travis, T Hibi, T Hisamatsu, D Fisher, M Shan, T H Gibble, D Rubin, J Jones

**Affiliations:** 1 University of Oxford, Oxford, United Kingdom; 2 Center for Advanced IBD Research and Treatment, Kitasato University Kitasato Institute Hospital; 3 Department of Gastroenterology and Hepatology, Kyorin University School of Medicine, Tokyo, Japan; 4 Eli Lilly and Company, Indianapolis; 5 University of Chicago Medicine Inflammatory Bowel Disease Center, Chicago, United States; 6 Department of Medicine, Department of Community Health and Epidemiology, Dalhousie University, Halifax, Canada

## Abstract

**Background:**

Bowel urgency (BU) was assessed in mirikizumab (miri) Phase 3 LUCENT studies in moderately-to-severely active UC using the validated Urgency Numeric Rating Scale (UNRS). UNRS measures BU severity in the past 24 hours from 0 (no urgency) to 10 (worst possible urgency). Psychometric evaluation of the UNRS showed Clinically Meaningful Improvement (CMI) is >3 point change; Remission is a score of 0 or 1.

**Purpose:**

This analysis evaluated the proportions of patients in LUCENT studies achieving BU CMI and BU remission.

**Method:**

The modified intent-to-treat (mITT) population (patients receiving ≥1 dose of miri or placebo (PBO); N= 1281) was randomized at induction study baseline in a 3:1 ratio to IV doses of 300mg miri or PBO every 4 weeks (Q4W) during induction (W0, 4, and 8). Patients achieving Clinical Response, measured by Modified Mayo Score (MMS), to miri during induction were re-randomized at W0 of the maintenance study in a 2:1 ratio to subcutaneous (SC) 200mg miri or PBO Q4W through W40 (52 weeks of treatment). Patients recorded their UNRS score daily in an e-diary. Mean weekly UNRS scores were calculated from diary data if ≥4 days of data were available. Rates of BU CMI and BU remission in the miri v PBO groups were compared at W12 (induction) in the mITT population with a baseline UNRS score ≥3, and W52 (maintenance) among miri clinical responders at W12 with a baseline UNRS score ≥3. Cochran-Mantel-Haenszel tests with non-responder imputation for missing values were used for all treatment comparisons.

**Result(s):**

Patient population: mean age 43 years, 60% male, disease duration 7 years; 63.0% left-sided colitis; 36.3% pancolitis; 46.7% moderate disease (MMS 4-6); 53.2% severe disease (MMS 7-9). Significantly higher proportions of miri versus PBO patients achieved BU CMI (48.7% v 32.2%) and BU remission (22.1% v 12.3%) at W12 (both p<0.001; Table) in the induction study. Similarly, at W40 of maintenance, significantly greater proportion of miri patients achieved BU CMI (65.2% v 41.9%) and BU remission (42.9% v 25.0%) compared to PBO among miri induction responders (both p<0.001; Table).

**Image:**

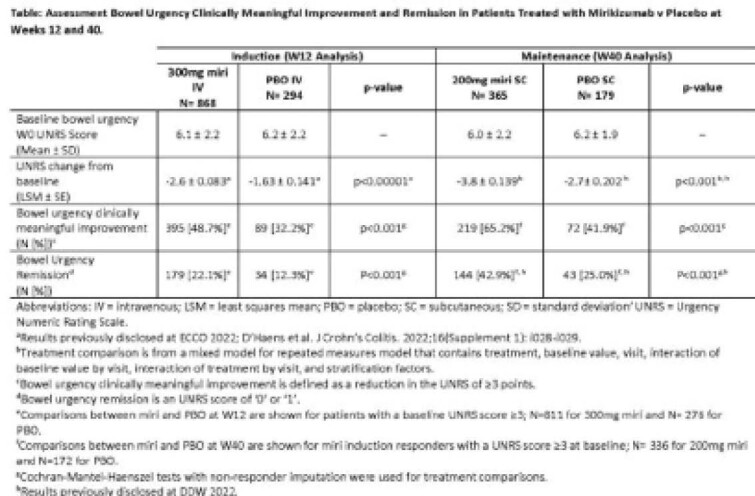

**Conclusion(s):**

Miri had a highly significant and clinically meaningful benefit on reducing bowel urgency, one of the most disruptive UC symptoms. The Urgency Numeric Rating Scale usefully quantified the baseline level and change in bowel urgency after treatment across a spectrum of severity.

**Please acknowledge all funding agencies by checking the applicable boxes below:**

Other

**Please indicate your source of funding;:**

Eli Lilly and Company

**Disclosure of Interest:**

None Declared

